# Supplementary material for: In vitro biofilm formation by a beneficial bacterium partially predicts in planta protection against rhizosphere pathogens
Source: ISME J. 2025 Jun 2;20(1):wraf114. doi: 10.1093/ismejo/wraf114 (PMC12919676; doi:10.1093/ismejo/wraf114)
Supplement: Supplemental_Materials_wraf114 [file supplemental_materials_wraf114.pdf]

Supplemental figures and table

In vitro biofilm formation by a beneficial bacterium partially predicts *in planta* protection against rhizosphere pathogens

Yang Liu*, Alexandra D. Gates*, Zhexian Liu, Quinn Duque, Sierra S. Schmidt, Melissa Y. Chen, Corri D. Hamilton, George A. O'Toole, and Cara H. Haney

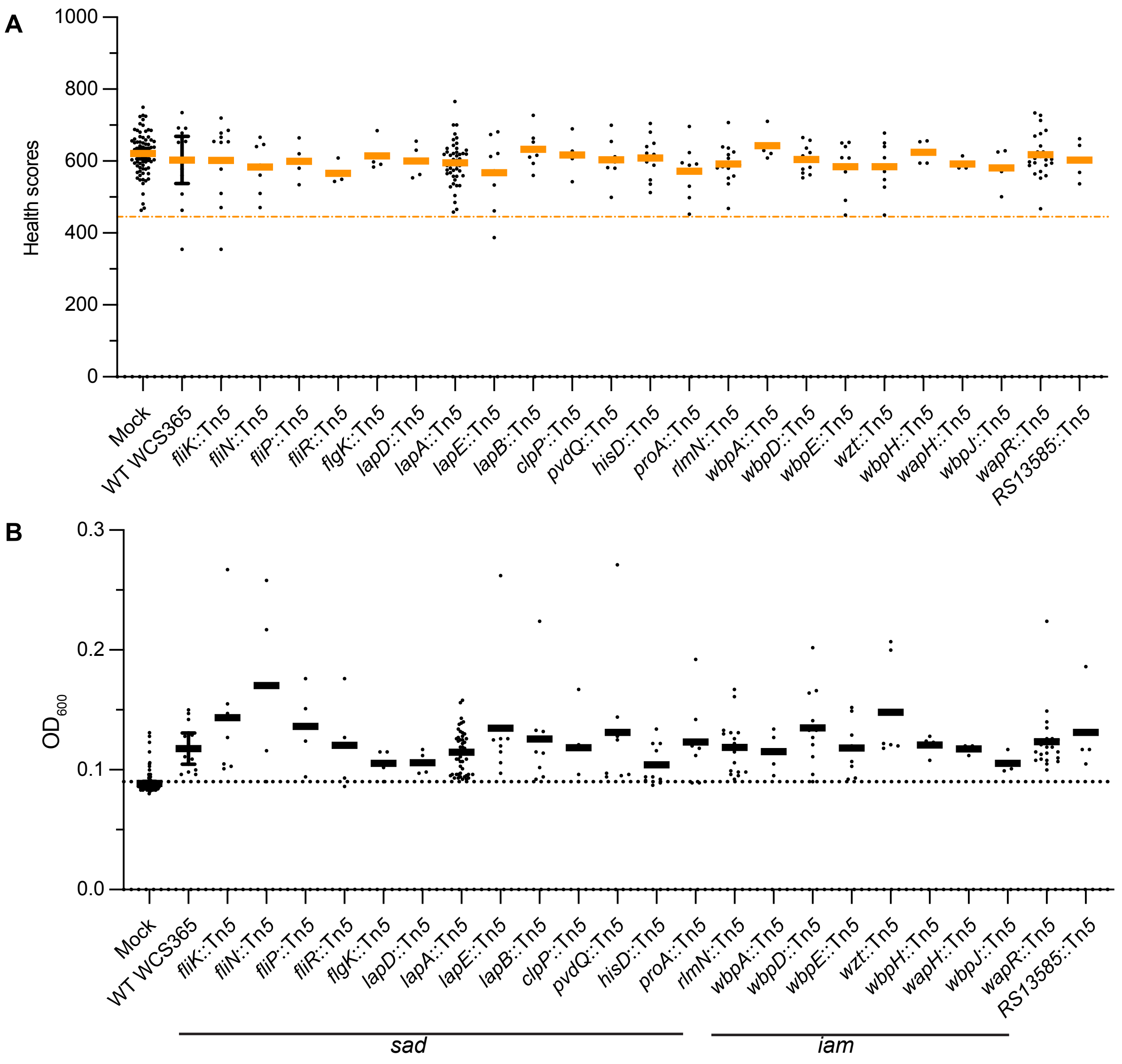

Figure S1. *P. brassicacearum* WCS365 *iam* and *sad* mutants do not affect plant health in mono association. A) Seedlings grown in MYCroplanters were treated with the *iam* and *sad* libraries in the absence of pathogen and plant health was quantified from scanned images. B) OD_{600} was measured for each mutant when grown in association with plants. A-B) Each point represents a single plant; lines represent the mean, and error bars for Mock and WT WCS365 represent 95% confidence intervals.

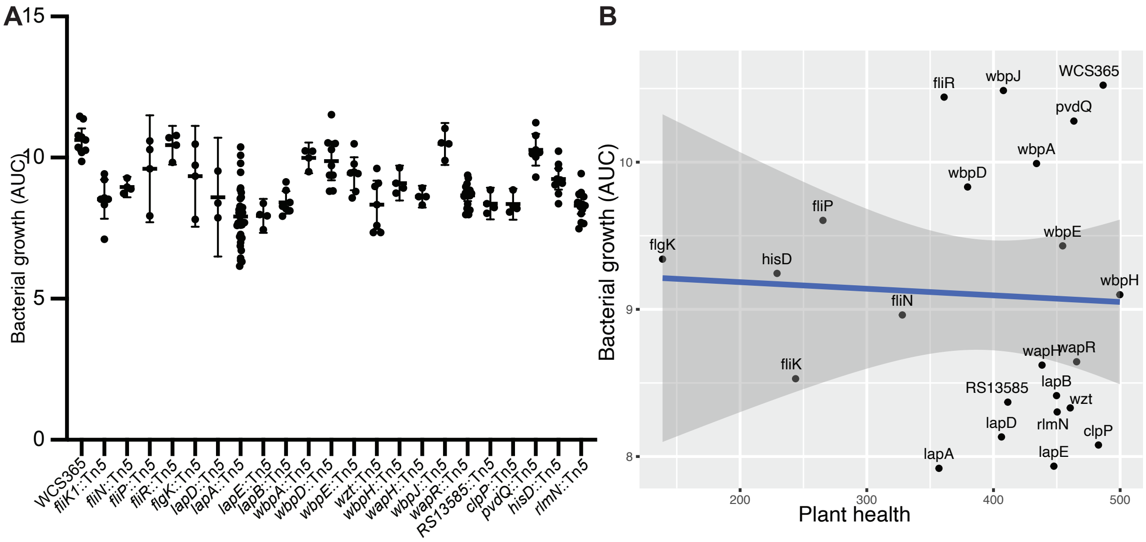

Figure S2. ***In vitro*** **and** ***in planta*** **growth of mutants**. A) Transposon insertion mutants were grown *in vitro* in LB medium and the area under the curve was quantified. B) A linear regression was performed between the ability to protect plants from pathogens and *in vitro* bacterial growth and no significant correlation was noted.

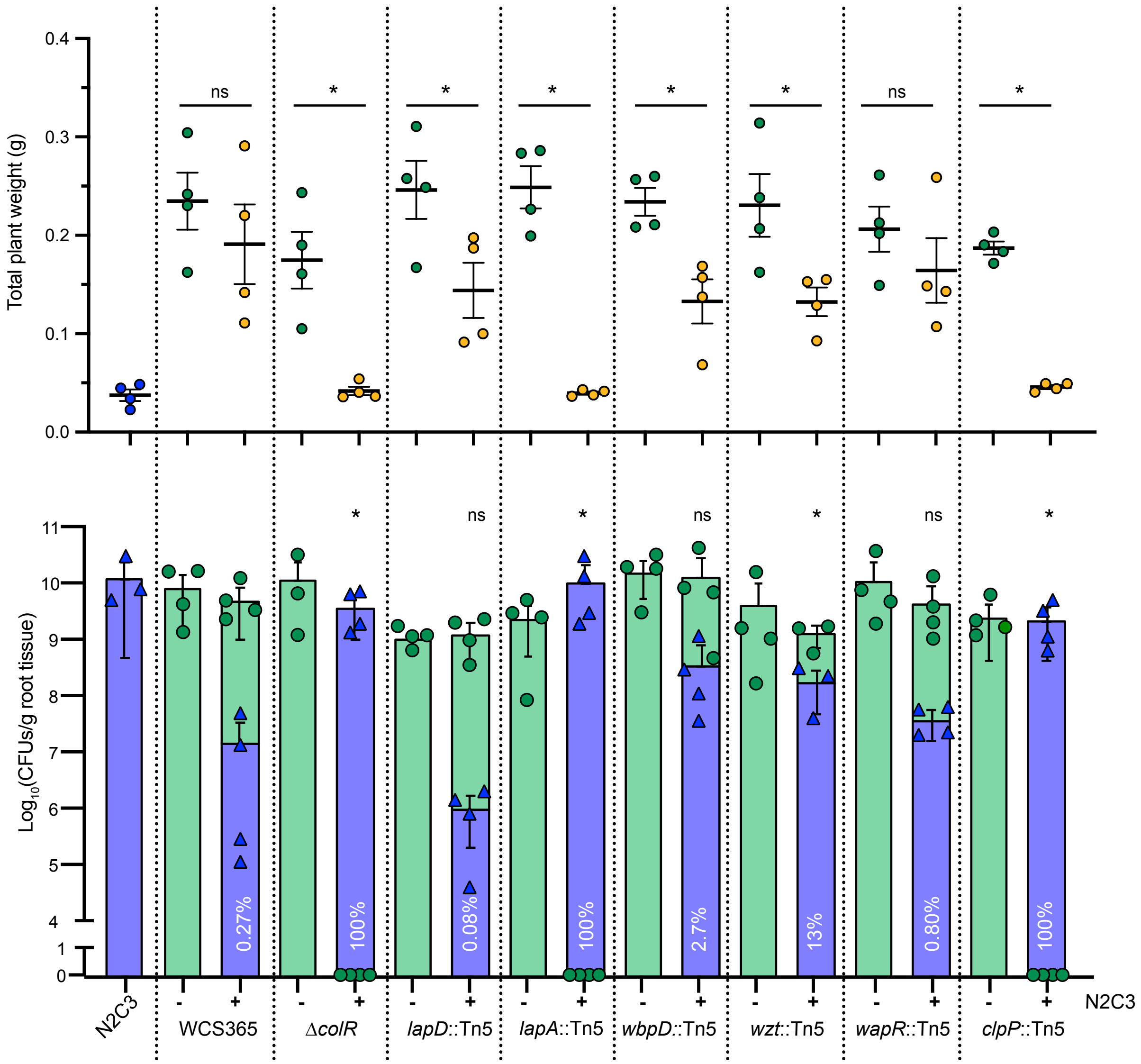

Figure S3. Quantification of bacterial competition at the rhizoplane shows some nonprotective WCS365 biofilm mutants are outcompeted by the N2C3 pathogen while others can co-exist. A) Plant weight (from 3 pooled plants weighed together) in protection assays with *iam* and *sad* mutants co-inoculated with N2C3 expressing *lacZ.* B) To quantify both the *iam* and *sad* mutants and N2C3 abundance in the rhizosphere, roots were washed and ground to quantify CFUs. Plotted on a $\log_{10}$ scale, N2C3 abundance is shown in blue triangles and WCS365 and *iam* and *sad* mutants are shown in green circles. Numbers indicate the fraction of the community that is N2C3 and indicates N2C3 abundance that is significantly enriched ($P <$ 0.05) relative to N2C3 in competition with wildtype bacteria by ANOVA and Tukey's HSD.

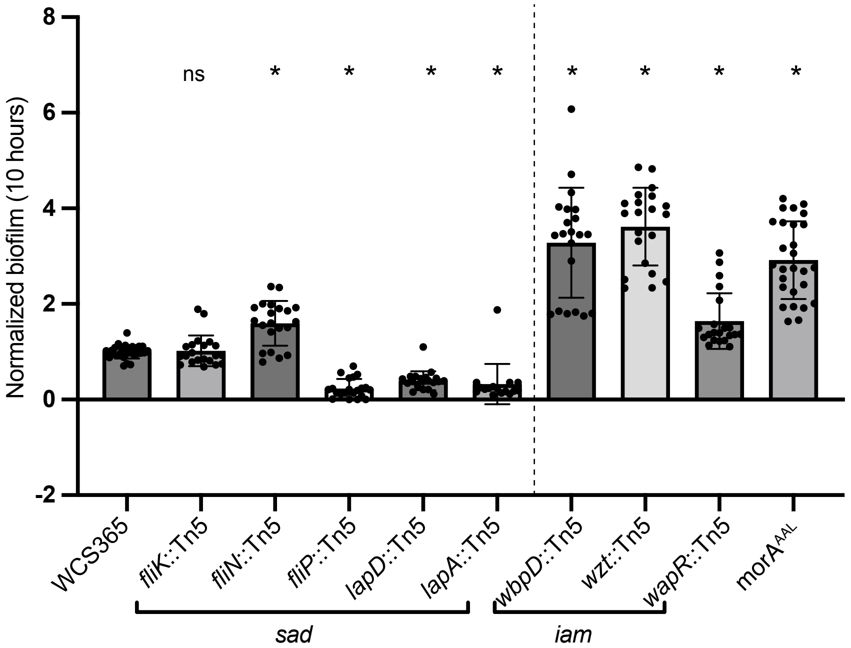

Figure S4. Biofilm formation at 10hrs shows similar patterns as at 18 hrs. To test if discrepancies in biofilm formation in our study compared to the original description of flagellar mutants as decreased attachment was due to the longer incubation time, we repeated biofilm assays for a subset of mutants at 10 hours. Those with biofilm formation that is statistically significant from wildtype ($P < 0.05$; Tukey's HSD) are indicated with an asterisk; ns indicates non-significant differences.

Table S1. Insertion sites of transposon insertions in the *iam* and *sad* libraries

Original Mutant name	Well	Seq published	Gene name	Locus Tag	Gene position	Figure 3
sad-97::Tn5-B22 (Gm, 'lacZ)	F7	This study	*moaA*	LRP86_RS02125	799(969)	No
sad-46::Tn5B30(Tc)	D8	This study	*mexW*	LRP86_RS03915	1709(3027)	No
sad-101::Tn5-B22 (Gm, 'lacZ)	F10	This study	*sadC*	LRP86_RS04535	184(1107)	Yes
sad-63::Tn5-B22 (Gm, 'lacZ)	E7	This study	*HP5*	LRP86_RS05960	1273(1725)	No
sad-58::Tn5-B22 (Gm, 'lacZ)	E5	This study	*morA*	LRP86_RS06630	4245(4248)	No
iam-11::Tn5Gent	A10	This study	*wbpJ*	LRP86_RS06850	216(1227)	Yes
iam-5::Tn5Gent	A4	This study	*wbpH1*	LRP86_RS06855	449(1242)	No
iam-2::Tn5Gent	A2	This study	*wapH2*	LRP86_RS06880	2273(3243)	Yes
iam-22::Tn5B30(Tc)	A12	This study	*wzt1*	LRP86_RS06885	204(1785)	Yes
iam-22::Tn5B30(Tc)	B2	This study	*wzt1*	LRP86_RS06885	204(1785)	No
iam-21::Tn5B30(Tc)	A11	This study	*wbpE1*	LRP86_RS06900	372(1092)	No
iam-21::Tn5B30(Tc)	B1	This study	*wbpE2*	LRP86_RS06900	372(1092)	Yes
iam-23::Tn5B30(Tc)	B3	This study	*wbpD1*	LRP86_RS06905	508(585)	Yes
iam-25::Tn5B30(Tc)	B7	This study	*wbpD2*	LRP86_RS06905	508(585)	No
iam-26::Tn5B30(Tc)	B8	This study	*wbpD3*	LRP86_RS06905	508(585)	No
iam-24::Tn5B30(Tc)	B4	This study	*wbpA*	LRP86_RS06915	592(1309)	Yes
sad-12::Tn5B30(Tc)	C6	This study	*proA1*	LRP86_RS07155	82(1272)	No
sad-98::Tn5-B22 (Gm, 'lacZ)	F8	This study	*proA2*	LRP86_RS07155	264(1272)	No
sad-45::Tn5B30(Tc)	D7	This study	*bioF*	LRP86_RS08345	137(1179)	No
sad-102::Tn5-B22 (Gm, 'lacZ)	F11	This study	*metF*	LRP86_RS08870	intergenic	No
sad-10::Tn5B30(Tc)	C4	This study	*HP1*	LRP86_RS10235	1323(1415)	No
sad-19::Tn5B30(Tc)	D1	O'Toole 2006	*lapD*	LRP86_RS12025	1470(1947)	Yes
sad-8::Tn5Gent	C2	This study	*lapA1*	LRP86_RS12045	994(14239)	No
sad-81::Tn5-B22 (Gm, 'lacZ)	E10	This study	*lapA10*	LRP86_RS12045	11179(14239)	No
sad-82::Tn5-B22 (Gm, 'lacZ)	E11	This study	*lapA11*	LRP86_RS12045	11182(14239)	No
sad-86::Tn5-B22 (Gm, 'lacZ)	F2	This study	*lapA12*	LRP86_RS12045	4386(14239)	No
sad-9::Tn5Gent	C3	This study	*lapA2*	LRP86_RS12045	2755(14239)	No
sad-18::Tn5B30(Tc)	C12	O'Toole 1998	*lapA3*	LRP86_RS12045	6411(14239)	No
sad-47::Tn5B30(Tc)	D9	This study	*lapA4*	LRP86_RS12045	12307(14239)	No
sad-51::Tn5-B22 (Gm, 'lacZ)	D11	This study	*lapA5*	LRP86_RS12045	4385(14239)	No
sad-53::Tn5-B22 (Gm, 'lacZ)	E1	This study	*lapA6*	LRP86_RS12045	4136(14239)	No
sad-62::Tn5-B22 (Gm, 'lacZ)	E6	This study	*lapA7*	LRP86_RS12045	4136(14239)	No

sad-79::Tn5-B22 (Gm, 'lacZ)	E8	This study	*lapA8*	LRP86_RS12045	11171(14139)	Yes
sad-80::Tn5-B22 (Gm, 'lacZ)	E9	This study	*lapA9*	LRP86_RS12045	11261(14239)	No
sad-7::Tn5Gent	C1	This study	*lapE*	LRP86_RS12055	987(1350)	Yes
sad-84::Tn5-B22 (Gm, 'lacZ)	F1	This study	*lapE*	LRP86_RS12055	1241(1350)	No
sad-52::Tn5-B22 (Gm, 'lacZ)	D12	This study	*lapB*	LRP86_RS12060	1095(2157)	Yes
sad-87::Tn5-B22 (Gm, 'lacZ)	F3	This study	*lapB*	LRP86_RS12060	1744(2157)	No
iam-8::Tn5Gent	A7	This study	*LRP86_RS13585*	LRP86_RS13585	667(1142)	Yes
iam-1::Tn5Gent	A1	This study	*wapR1*	LRP86_RS13590	696(885)	Yes
iam-3::Tn5Gent	A3	This study	*wapR2*	LRP86_RS13590	676(885)	No
iam-6::Tn5Gent	A5	This study	*wapR3*	LRP86_RS13590	697(885)	No
iam-7::Tn5Gent	A6	This study	*wapR4*	LRP86_RS13590	688(885)	No
iam-9::Tn5Gent	A8	This study	*wapR5*	LRP86_RS13590	697(824)	No
iam-10::Tn5Gent	A9	This study	*wapR6*	LRP86_RS13590	676(885)	No
sad-89::Tn5-B22 (Gm, 'lacZ)	F4	This study	*hisD*	LRP86_RS15545	443(1338)	No
sad-95::Tn5-B22 (Gm, 'lacZ)	F5	This study	*hisD*	LRP86_RS15545	442(1338)	No
sad-96::Tn5-B22 (Gm, 'lacZ)	F6	This study	*hisD*	LRP86_RS15545	442(1338)	No
sad-55::Tn5-B22 (Gm, 'lacZ)	E2	This study	*rlmN1*	LRP86_RS15910	1106(1149)	No
sad-56::Tn5-B22 (Gm, 'lacZ)	E3	This study	*rlmN2*	LRP86_RS15910	956(1149)	No
sad-57::Tn5-B22 (Gm, 'lacZ)	E4	This study	*rlmN3*	LRP86_RS15910	1113(1149)	No
sad-100::Tn5-B22 (Gm, 'lacZ)	F9	This study	*rlmN4*	LRP86_RS15910	911(1149)	No
sad-14::Tn5B30(Tc)	C8	O'Toole 1998	*flgK1*	LRP86_RS18635	255(2016)	Yes
sad-16::Tn5B30(Tc)	C10	This study	*fliK2*	LRP86_RS18735	307(1374)	Yes
sad-17::Tn5B30(Tc)	C11	This study	*fliK*	LRP86_RS18735	371(1374)	No
sad-20::Tn5B30(Tc)	D2	This study	*fliN*	LRP86_RS18750	299(459)	Yes
sad-13::Tn5B30(Tc)	C7	O'Toole 1998	*fliP*	LRP86_RS18760	403(750)	Yes
sad-48::Tn5-B30 (Tc)	D10	This study	*fliR*	LRP86_RS18770	544(783)	Yes
sad-11::Tn5B30(Tc)	C5	O'Toole 1998	*clpP1*	LRP86_RS20465	29(636)	Yes
sad-83::Tn5-B22 (Gm, 'lacZ)	E12	This study	*rpfC*	LRP86_RS23115	828(1638)	Yes
sad-22::Tn5B30(Tc)	D4	This study	*HP2*	LRP86_RS23120	-54(498)	No
sad-44::Tn5B30(Tc)	D6	This study	*HP4*	LRP86_RS23825	314(627)	No
sad-15::Tn5B30(Tc)	C9	This study	*pvdQ1*	LRP86_RS25805	385(2349)	Yes
sad-21::Tn5B30(Tc)	D3	This study	*pvdQ2*	LRP86_RS25805	369(2349)	No
sad-43::Tn5B30(Tc)	D5	This study	*HP3*	no hits		No
